# Insights on Hysteroscopic Procedures and Their Place in Romanian Gynecologic Practice—The Experience of Two Medical Units

**DOI:** 10.3390/diagnostics10050281

**Published:** 2020-05-06

**Authors:** Alexandra Matei, Cringu Ionescu, Florin Gorun, Diana Gheorghiu, George-Alexandru Rosu, Adelina Dan, Romina-Marina Sima, Cristian George Furau, Corina Ilinca, Dan Navolan

**Affiliations:** 1 Department of Obstetrics and Gynecology, “Carol Davila” University of Medicine and Pharmacy, Dionisie Lupu Street, no. 37, 030167 Bucharest, Romania; ale.matei@gmail.com (A.M.); george.rosu@ymail.com (G.-A.R.); danadelina90@gmail.com (A.D.); romina.sima@yahoo.es (R.-M.S.); 2 Department of Obstetrics and Gynecology, “Victor Babes” University of Medicine and Pharmacy, Eftimie Murgu Square, no. 2, 300041 Timisoara, Romania; lorin.gorun@yahoo.com (F.G.); navolan@yahoo.com (D.N.); 3 Department of Obstetrics and Gynecology, “St. Pantelimon” Emergency Hospital, Pantelimon Blvd, no. 340-342, 021659 Bucharest, Romania; cdgheorghiu@hotmail.com; 4 Department of Obstetrics and Gynecology, Emergency County Hospital, Andreny Karoly Street, no. 2-4, 310037 Arad, Romania; cristianfurau@gmail.com; 5 Faculty of Sociology and Social Work and Statistical Office, University of Bucharest, Panduri Street, no. 90, 050663 Bucharest, Romania; bragaru_corina@yahoo.com

**Keywords:** hysteroscopy, endometrial hyperplasia, myoma, isthmocele, infertility

## Abstract

Hysteroscopy has known an increasing use in Romania over the last decade, succeeding to mark an impact on lowering the costs of medical services. The strategy of this study was to present the experience of two Romanian medical units with different experiences using inpatient regimen hysteroscopy, and to further compare it with current worldwide tendencies. Strong points in our practice were sought, as well as components that require improvement. Overall, abnormal uterine bleeding stands for most hospital case presentations in our study group; hysteroscopy had the highest accuracy and positive predictive value in identifying uterine myomas: 91.03% and 100%, respectively; for endometrial polyps, statistical analysis showed the highest sensitivity: 100%, with 83.89% specificity and a 77.64% positive predictive value. The applicability of hysteroscopy was further described for infertility cases and isthmocele repair.

## 1. Introduction

The Romanian health care system is currently situated at a threshold marked by worldwide political fluctuations, which mostly converge on influencing the availability of economic supplies. Statistics on hospital discharges and the average length of hospital stays reflect the balance between the demand for and supply of hospital services; in 2017 the average length of hospital stay for in-patients was 7.5 days in Romania, while in Hungary in-patients spent in average of 9.8 days in hospital, at the opposite end of the scale, the Netherlands recorded an average of 4.5 days length of hospital stay [1,2]. Gynaecological practice—and particularly patients diagnosed with pathologies who could benefit either from minimally invasive or conservatory treatment options—are most favoured by the aforementioned data.

With 74%–100% sensitivity and 93%–99% specificity in diagnosing benign uterine pathologies, hysteroscopy can assure fast track diagnostic setup, with a one stop see-and-treat service [3,4]. Experience in our country is, however, heterogeneous, since the equipment is not ubiquitous in territorial medical units.

To address this issue, this present study was developed in two medical centers and aimed to comprise both the role of hysteroscopy in confirming the correlation between clinical suspicion and histopathological (HP) diagnosis, and also its advantages and limitations as a therapeutic procedure. We chose to present our analysis on each center in a separate manner, in order to underline mainly the impact of minimally invasive procedures on medical service performed in second versus third degree units; also, the two hospitals are located in different areas of the country, having different patient addressability regarding both patient number and medical conditions: in Timisoara city there are only two specialty medical units, while in Bucharest there are nine. 

## 2. Materials and Methods 

We conducted a retrospective study in 2 Romanian medical centers: “St. Pantelimon” Emergency Hospital—a tertiary care unit from Bucharest—and Timisoara Municipal Clinical Hospital—a secondary territorial medical care unit. 

The strategy we considered for this study originated in the comparison of medical practice using hysteroscopy equipment in either diagnosis or treatment procedures performed in the hospitals mentioned above, by underlining the advantages and limitations of its use, and further projecting data in the current medical trends framework. 

Patient electronic charts and surgical protocol registries were used as source information on hysteroscopic procedures performed in both centers. 

Patient inclusion criteria were as follows:

- Women aged over 18 years;
- Admitted to “St. Pantelimon” Emergency Hospital during January 2014–October 2019 or at Timisoara Municipal Clinical Hospital during October 2018–October 2019;
- Underwent hysteroscopic procedure.

The established exclusion criteria were patients under 18 years old who did not have a hysteroscopic procedure performed during hospitalization. 

Patients included in the study had hysteroscopies performed using Karl Storz Endoscopy Aida Control Tutlingen Germany rigid hysteroscope. Demographic data regarding patient age, urban or rural background, menopausal status, as well as comorbidities, were collected. Specifically, clinical suspicion diagnosis, hysteroscopic diagnosis and procedures along with corresponding HP diagnosis were gathered in a database. We have considered rural settings the smallest administrative and territorial units named villages, as well as groups of villages named communes—larger living areas we have considered as urban settings. 

Microsoft Office 365 Excel was used to statistically analyze the information. First, we used descriptive statistics to underline patient health profile and background; Second, for nominal variables like hysteroscopy indication and procedures, we considered chi square test in order to verify if the two-unit results are due to chance or not. Hysteroscopy examination characteristics: sensitivity, specificity, positive predictive value and accuracy were also determined, based on HP diagnoses; Last, the Kolmogorov-Smirnoff test was used to verify the normal distribution of continuous variables like patient age, while one tail t-test was applied on the same data to verify if there was any significant age difference between patients diagnosed with simple versus complex endometrial hyperplasia. 

Patients included in this report were not provided any written consent due to the retrospective nature of this study. This study was approved by the Ethic Committees of each medical unit involved: reg no. 4340/2020, 12529/2020).

## 3. Results

From “St. Pantelimon” Emergency Hospital, there were 45 patients included in the study. The mean age of participants was 39 years old. Most patients (88.88%) were based in urban areas of residence; 95.56% of women were in their reproductive period of life while 4.44% were menopausal.

From Timisoara Municipal Clinical Hospital there were 323 reported hysteroscopies during the study period; the mean age of patients was 41 years old. Regarding the residential area of patients, most of them (63.8%) came from urban settings. Menopausal status was recorded and is depicted as follows: 80.45% of females were in their fertile period of life, while 19.55% were in menopause. 

**Figure 1:**
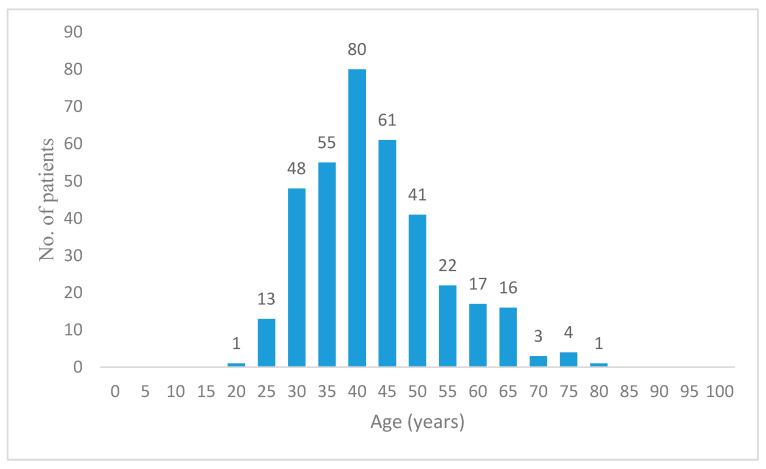
Patient distribution according to age groups.

The difference between the number of patients attending these units is generated by the fact that in Bucharest there are nine available third level specialty clinics, while in Timisoara are only two. Demographic data on patients from each center and cumulative descriptive statistics are presented in Table 1. Patient distribution according to age groups is depicted in Figure 1. 

Based on the admission diagnosis established by clinical and ultrasound evaluation, hysteroscopy indications were as follows: in Bucharest centre, 80.00% of patients complained of abnormal uterine bleeding (AUB), this being the most frequent indication for hysteroscopic diagnosis; one patient (2.22%) had postmenopausal bleeding; the presumptive diagnosis of endometrial and cervical polyps accounted for 55.55% of cases, and justified the procedure. The third most frequent indication was myoma diagnosis in 40.00% of women included in the study. Hysteroscopy was also used as diagnostic tool in patients with secondary infertility (11.11%) and chronic pelvic pain (6.66%). Compared to this medical framework, in Timisoara hospital, AUB was responsible for only 29.41% of the presenting cases, of which 4.33% were postmenopausal; the main reason for hysteroscopic evaluation was suspicion of endometrial and cervical polyp (39.62%), followed by uterine myoma (11.45%) and primary infertility (10.52%). Other presumptive diagnoses which recommended hysteroscopy evaluation were related to endometrial structural pathologies: suspected endometrial hyperplasia, comprising of 15.55% of cases in Bucharest and 5.26% of cases in Timisoara. Intrauterine device (IUD) retention required 5.88% of hysteroscopic interventions in Timisoara. Detailed information on indications for hysteroscopic evaluation are further presented in Table 2. Also, we found that hysteroscopy indications in the two centres are significantly different (*p* = 0.006, α = 0.05, chi square test for independence).

After uterine cavity exploration, the hysteroscopic diagnosis confirmed significant involvement of endometrium in causing AUB, as seen in Table 3: in Bucharest centre, polyps were observed in 37.77% of situations, and thickening of endometrial mucosa in 35.55% of cases. Uterine myomas were noted in 13.33% of cases, but only 6.66% were described as intracavitary nodules. In Timisoara clinic, postprocedural conclusions recorded the same three pathologies: endometrial polyp was the most frequent entity encountered (47.36% of patients), followed by thick endometrial mucosa (14.55%) and uterine myomas (11.45%, of which 4.95% were intracavitary). Only 16 out of 19 suspected IUD retentions were confirmed, and in 10.52% of patients uterine synechia were identified. 

**Figure 2:**
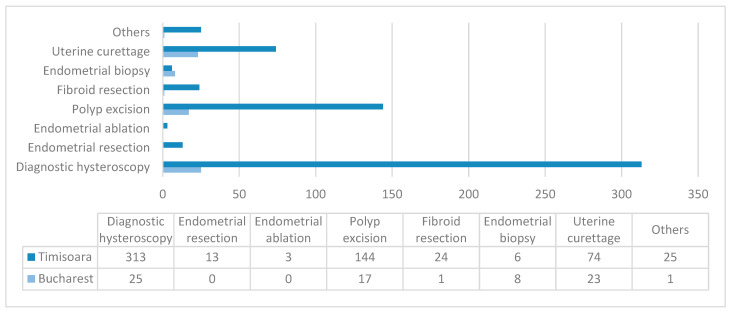
Main hysteroscopic procedures in the two centers. Data are presented as *N*.

Hysteroscopies were registered as diagnostic in 55.55% of cases in Bucharest and 96.90% of cases in Timisoara. Regarding the performed procedures in Timisoara, polyp excision and fibroid resection were most often conducted (44.58% and 7.43%, respectively). In Bucharest, however, results were slightly different, meaning that except from polyp excision in 37.77% of cases, in 17.77% of cases endometrial biopsy was carried and fibroid resection was only done in 2.22% of situations. Data analysis showed that dilation and curettage (D&C) is still a common practice in Gynaecology: it completed hysteroscopy investigation in 51.11% of cases in Bucharest and 22.91% in Timisoara, as seen in Table 4. A graphic representation of main hysteroscopic procedures can be found in Figure 2. A statistical chi square test for nominal variables showed that hysteroscopy procedures performed in Bucharest hospital were significantly different compared to those performed in Timisoara unit (*p* = 0.0001, α = 0.05).

HP examination offered the certainty diagnosis and helped identify the aetiology of bleeding, pelvic pain or infertility, as well as the subjacent pathophysiology (Table 5). Polyps were confirmed in all 46.66% cases in Bucharest, but in only 36.53% of cases from Timisoara. In detail, histologic description of endometrium concluded that beside secretory and proliferative stages of endometrium, elements of endometritis or different types of endometrial hyperplasia can have AUB as a primary label. Simple endometrial hyperplasia had the highest incidence (47.36% of cases in Timisoara and 6.66% of cases in Bucharest). The only cases of atypia correlated with endometrial hyperplasia were registered in Timisoara: A total of 3.09% of cases were associated with complex hyperplasia and 0.92% with simple hyperplasia; the reduced number of patients from the Bucharest unit is a possible reason that can explain the difference between the two centres. 

**Figure 3:**
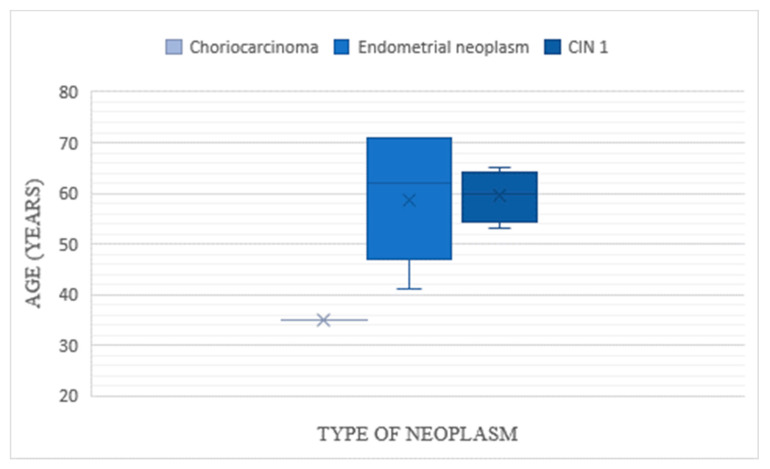
Distribution of patients with histopathological identified neoplasms according to age.

**Figure 4:**
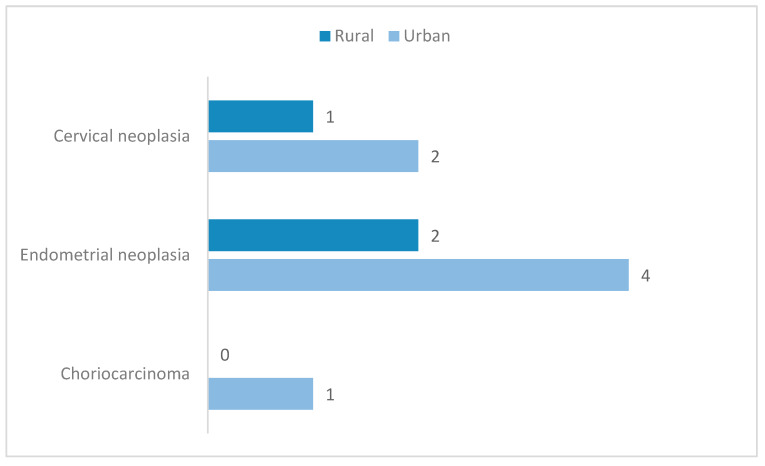
Neoplasia histopathological diagnoses distribution on patient background. Data are presented as *N*.

All aside, the aim of HP evaluation was primarily to determine or exclude malignancies. In our study, results showed that there was a total of 12 cases of neoplasms discovered after HP examination, and only one had been previously suspected during hysteroscopy. The Timisoara database marked the highest incidence of neoplasms in the study: six patients were diagnosed with endometrial malignancy, four with cervical neoplasm and one with choriocarcinoma. There was only one case of endometrial neoplasm identified in the Bucharest group, which was not previously suspected during hysteroscopic evaluation. The mean age of patients with cervical cancer was slightly higher than that of patients diagnosed with endometrial cancer (59.5 years vs 58.5 years), as seen in Figure 3. Additionally, the incidence of both types of malignancies was found to be twice higher in urban areas compared to rural ones (Figure 4); nevertheless, this result is not statistically significant (*p* = 1, α = 0.05, chi square test for independence).

All cases of cervical malignancy had simple dysplasia described as cervical intraepithelial neoplasm (CIN) 1. On the other hand, a HP exam described all cases of endometrial malignancy from Timisoara unit as endometrioid adenocarcinoma: two cases were grade 1 (G1), known as well differentiated, three cases were grade 2 (G2), or moderately differentiated and one case was grade 3 (G3), or poorly differentiated. Hysteroscopy suspected one case of endometrial adenocarcinoma that was not confirmed at HP evaluation; conversely, the six patients diagnosed with endometrial neoplasia had non-specific hysteroscopic aspects of endometrial hypertrophy (four cases), polypoid mass (one case) or pyometra (one case).

Comparing all data from both centres on hysteroscopic diagnoses versus HP certainty diagnoses focusing on the three most frequent pathologies identified in our study, we found that hysteroscopy had the highest accuracy and positive predictive value in identifying uterine myomas: 91.03% and 100%, respectively; for endometrial polyps statistical analysis showed the highest sensitivity: 100%, with 83.89% specificity and 77.64% positive predictive value. On the opposite end of the scale, hysteroscopy had the lowest accuracy in diagnosing endometrial hyperplasia: 61.95%, as well as the lowest sensitivity: 30.69%.

**Figure 5:**
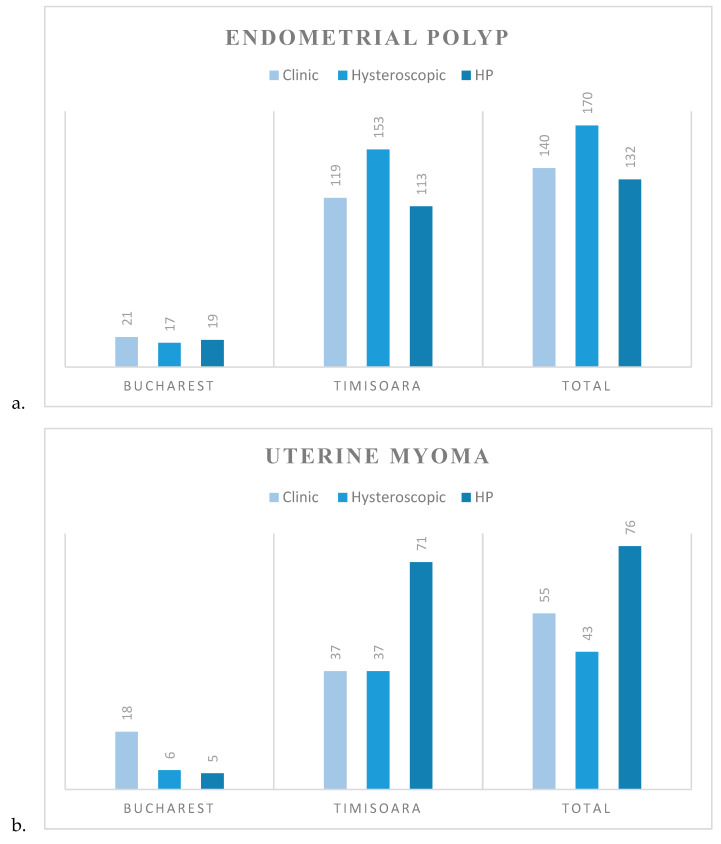

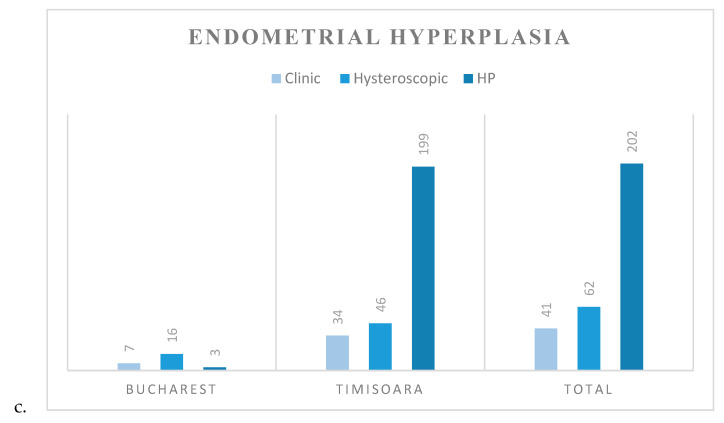
Comparison between clinical, hysteroscopic and histopathological diagnoses in each center and total evaluation for: (**a**) endometrial polyp; (**b**) uterine myoma; (**c**) endometrial hyperplasia. Data are presented as *N*.

Clinical, hysteroscopic and HP diagnoses on the aforementioned entities are further detailed in Figure 5.

Of 68 patients from the study group that were diagnosed with infertility (7 in Bucharest and 61 in Timisoara), 27 (39.70%: 4 from Bucharest and 23 from Timisoara) had polypoid mass identified during hysteroscopic diagnosis, but only 21 (30.88%: 4 from Bucharest and 17 from Timisoara) were further histologically diagnosed as endometrial polyps. Uterine myomas were also identified in patients from Timisoara with infertility: four cases (10.52%) identified by hysteroscopy and three (4.41%) confirmed histologically. 

**Figure 6:**
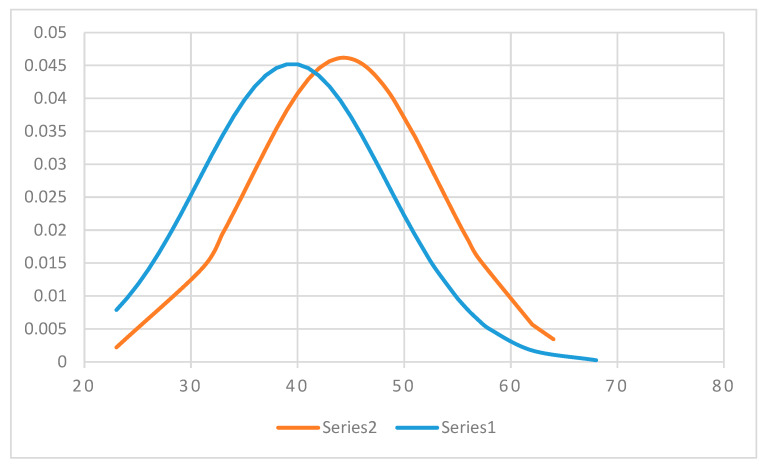
Simple (Series 1) and complex (Series 2) endometrial hyperplasia histopathological (HP) diagnosis distribution, according to patient age

As Figure 6 shows, the mean age of patients diagnosed with complex endometrial hyperplasia is significantly higher than that of patients diagnosed with simple endometrial hyperplasia: 44.30 years vs 39.48 years (*p* = 0.001, *t*-Test one tail). Both series of data have a Gaussian distribution verified with a Kolmogorov-Smirnoff test: for simple endometrial hyperplasia: *p* = 0.052, critical value = 0.110, α = 0.05; for complex endometrial hyperplasia: *p* = 0.094, critical value = 0.210, α = 0.05. 

**Figure 7:**
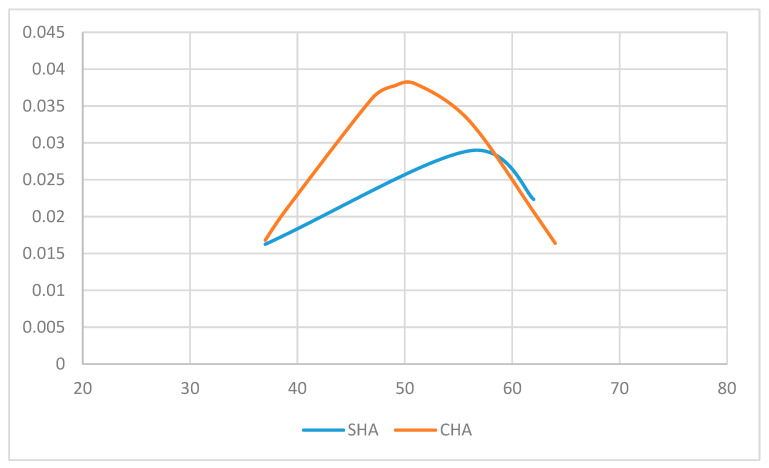
Simple (SHA) and complex (CHA) endometrial hyperplasia with atypia histopathologic diagnosis distribution, according to patient age

On the contrary, simple and complex endometrial hyperplasia with atypia HP diagnosis distribution according to patient age (Figure 7) showed a mean age of 51.66 years for patients with simple hyperplasia, compared to 50.40 years for patients with complex hyperplasia; the difference between the two age groups is not statistically significant: *p* = 0.44 (*t*-Test one tail). For this set of data, there was also a normal distribution of variables—the Kolmogorov-Smirnoff test—for simple endometrial hyperplasia with atypia: *p* = 0.296, critical value = 0.707, α = 0.05, and for complex endometrial hyperplasia with atypia: *p* = 0.165, critical value = 0.409, α = 0.05.

An additional analysis was carried out, in order to identify patients with anemia attributed to iron deficiency caused by AUB. We took into consideration the cutoff value of hemoglobin 12 g/dL proposed by World Health Organization (WHO). There were 32 (9.90%) patients from Timisoara who had hemoglobin values under 12 g/dL (minimum value of 6.72 g/dL), of whom only one had transitioned to menopause.

## 4. Discussion

Romania is an upper-middle income country [5], and in this economical context hysteroscopy equipment is not reachable in every sanitary unit across our country. The prevalence of AUB among reproductive aged women varies between 3%–30% worldwide, as the International Federation of Gynecology and Obstetrics (FIGO) Committee for Menstrual Disorders states, but the universal applicability of nomenclature systems and classifications is nowadays an ongoing process [6]. In our study, AUB accounted for 35.59% of included women, irrespective of their menopausal status.

It has been shown that objective quantification of monthly menstrual blood loss is not cost-effective, thus, patient medical history plays a significant role in AUB diagnosis [7]. In order to provide a systematic approach on the matter of AUB, efforts are being made to implement in the general medical care the core classification system, according to the acronym PALM-COEIN (pronounced “pahm-koin”): polyp, adenomyosis, leiomyoma, malignancy and hyperplasia—coagulopathy, ovulatory dysfunction, endometrial, iatrogenic and not yet classified, as it was approved by FIGO Executive Board for nonpregnant, reproductive aged women [8,9]. We took the first step in analysing the PALM concept that could explain the high incidence of AUB in our study, by using results from hysteroscopy procedure registries. 

This report is confined only to hysteroscopies performed in inpatient settings using rigid hysteroscopes. No intervention related complications were identified. Uterine polyps accounted for most of admission diagnoses, which pleaded for hysteroscopic confirmation and treatment (55.55% in Bucharest, and 39.62% in Timisoara). In Bucharest and Timisoara centres, simple transvaginal ultrasound (TV-US) was performed prior to hysteroscopic management. Sonohysterography is an alternative method for endometrial polyp and submucosal myoma diagnosis (specificity reaching almost 100%), mainly due to its ability to evaluate in detail the latter; it might also find its meaningful place in practice in the evaluation of perimenopausal women in whom, due to erratic ovarian oestrogen production, endometrium might be difficult to assess using TV-US alone [10,11,12,13]; however, it was not performed in any of the units included in our study 

We identified the fact that indications for hysteroscopy, as well as the associated procedures, were significantly different in the two centres. These results reinforce the importance of patient background: social context, geographic area of living, economic status—in influencing patient health and medical addressability. Although the observed minimally invasive techniques had in view similar pathologies, quantitative analysis showed a marked difference between them. 

Uterine polyps are found in 10% of general female population, and in 27% of cases, spontaneous regression has been observed [10]. Patient satisfaction improvement, an evidenced high success rate of 75%–100% in terms of symptom relief after polyp resection, and the possibility to identify premalignant and malignant tissue changes, with a pooled prevalence of 0.3% and 8.1%, has determined Lieng et al. to recommend treatment for women diagnosed with endometrial polyps [14,15]. This is in accordance with our results, which show that polypectomy was performed for most patients previously suspected to have polypoid masses. 

In the era of assisted reproductive techniques, there were some worrisome questions regarding the interference of polyps—especially those found incidentally—with embryo implantation and therefore with poor pregnancy rates. With recorded incidence of 4% of all women with unexplained infertility and 14.8% of infertile women with eumenorrhea [11], endometrial polypectomy prior to infertility treatment has become a common practice. Hysteroscopic polypectomy improved spontaneous pregnancy rates up to 78.3%, compared to 42.1% in patients with a normal uterine cavity, as well as cumulative pregnancy rates, using intrauterine insemination up to 38.3%, compared to 18.3% (*p* = 0.015) in patients who did not undergo intervention [10]; the same benefit is supported by a Cochrane review as well [16]. In our study, almost 40% of patients diagnosed with infertility had hysteroscopic findings suggestive for polyps; moreover, almost 31% were confirmed at HP examination, showing that this pathology can indeed be responsible for the failure in achieving a pregnancy. Special attention should be directed towards uterine fibroids, since almost 11% were identified in infertile women. Follow up data on these patients regarding their pregnancy rates were absent, which could represent a possible drawback of one step “see and treat” hysteroscopy.

Related to leiomyomas, hysteroscopic evaluation managed to identify intracavitary myomas in 6.66% and 4.95% of cases in Bucharest and Timisoara, respectively, and fibroid resection followed in 2.22% and 7.43% of the cases, respectively. The difference between the Timisoara results reside in the fact that not all fibroids visualised during the procedure were properly described in relation to the uterine cavity. Since long term follow up studies reveal an increase of recurrences [17], complete nodule removal is mandatory. 

The D&C approach is nowadays considered a historic procedure, for uterine polyps only have a 4% chance of complete removal [17]. The overall 26.35% rate of D&C in our study group is explained by unavailability of certain hysteroscopic instruments, and possibly by the difficulty some physicians face in changing habits. Both reasons are ready to be surpassed in our clinics, and D&C rates are expected to decrease progressively. According to the 2019 revised National Guidelines, cervical cancer accounts for approximately 67% of genital cancers in Romania, while endometrial cancer is responsible for another 7.8%, thus justifying the assertive strategies clinicians implement in cases of AUB [18,19]. These statistics are also involved in the practice of defensive medicine in our country, with D&C offering an elusively reassuring method for the physician to exclude a possible malignancy. 

Treatment goals for patients with AUB should be focused on improving women’s quality of life [20], and it should be tailored to the patient, even in the case of adenomyosis, and particularly when women also wish to preserve fertility. In our study, adenomyosis was suspected in 1.63% of the cases after TV-US exam, while hysteroscopic exploration of the uterine cavity suspected this diagnosis in only 0.54% of the cases. HP diagnosis revealed that, in 7.06% of the cases, adenomyosis was present; from this perspective, TV-US is superior to hysteroscopy in detecting myometrial morphologic variations corresponding to adenomyosis. 

On the other hand, hysteroscopy is disclosed to have high accuracy in determining pre-malignant and malignant endometrial disease [9], but from our 64 menopausal women in the study group, only four had an endometrial biopsy during hysteroscopy. Taking into account that age alone is an independent risk factor for endometrial neoplasia, and that during menopause, the risk of malignancy is estimated to be 7% for polyps and 1% for myomas [20,21], we consider our proportion of 6.25% biopsied patients to be evocative of the need for an invariable guideline, destined to establish a customised menopausal patient care algorithm, even moreif we consider that, for older patients, hysteroscopic myomectomies have better outcomes [22]. 

The results we have obtained showing a low accuracy of 61.95% in diagnosing endometrial hyperplasia are representative of diagnostic hysteroscopy alone, directed endometrial biopsy being excluded. Our results are the opposite of a large randomized controlled trial, which showed that only in 0.9% of patients endometrial hyperplasia was missed during the diagnostic procedure [9].

In Timisoara, the incidence of neoplasia with the hysteroscopic appearance of polypoid mass reached 0.60% (one out of 165 cases); this is in accordance with data showing a 0.5%–3% endometrial polyp cancer prevalence [9]. The fact that, in our report, the incidence of cervical and endometrial cancers was twice as high as in urban settings compared to rural ones confirms previous findings on the fact that, nationally, the cases of endometrial cancer most frequently come from urban areas compared to rural areas [23]. 

We paid special attention to anemia as side effect of AUB, since WHO underlined the effects of iron deficiency anemia on the cognitive and motor skills of patients, as well as on causing a disruptive social behavior [24]. Almost 10% of patients from Timisoara had hemoglobin levels under 12 g/dL, and beside the relatively high incidence of this finding, an alarming fact is that except one woman all the others were in their fertile period of life. 

There was only one case of isthmocele hysteroscopic repair in our study but, nevertheless, with 19.4% to 84% prevalence among symptomatic patients with postmenstrual spotting as the main presenting symptom, this entity should be part of the differential diagnosis when evaluating patients with a history of caesarean section and AUB. Moreover, since Romanian obstetrics still represents a nucleus of caesarean section deliveries, in 2014, having the third highest rate in Europe of 38%, emerging mostly from defensive practice of medicine, as we previously discovered [25,26], a cautious investigational approach should be compulsory. Mainly when results of minimally invasive repair of the uterine defect on patient fertility are promising [27].

Beside investigating the aetiology of AUB, hysteroscopy was successfully performed in 4.34% cases of IUD retention, and 1.90% cases of uterine synechia lysis. The wide spectrum of applicability of this procedure has found its place both in the secondary and tertiary care units, proving to be an efficient method for diagnosis and treatment. 

The lack of specific and detailed pattern describing hysteroscopic procedures and data collection from patient charts and registries are weak points in our study that we struggled to overcome as potential source of errors. A detailed description of AUB diagnosis and postinterventional patient follow up are accounted for as points to be improved in our general practice. 

Hysteroscopy provides a valuable tool for the diagnosis and treatment of numerous uterine structural abnormalities. Its beneficial role in current medical practice resides not only in permitting patients to resume their activities immediately by rendering comfort, rapidity and practical management of gynecological drawbacks, but also by involving reduced invasiveness and costs. These aspects are essential especially for patients living in our society. 

## 5. Conclusions

Our study describes the circumstances in which hysteroscopy is applied in two representative medical units from Romania. Although successful in detecting the most frequent etiologies of AUB, hysteroscopy also has its limits: by presenting the framework of symptoms, presumptive diagnoses and small procedures performed in the clinics, we were able to identify the emerging need for an alternative imaging diagnosis tool, which is saline infusion sonography and its niche applicability. D&C is still a routine practice in Romania, but with the spread of hysteroscopic equipment which allows direct visualization of uterine cavity, the rates are expected to diminish. 

In both regions of our country, endometrial polyps, uterine myomas, adenomyosis and malignancies accounted for most presenting cases, as described by the PALM acronym. Compared to worldwide statistics, which are multifactorial and complex, we have managed to identify our communities from different regions of the country along these lines. 

## Figures and Tables

**Table 1:** Descriptive statistics of patient age, area of residence and menopausal status.

	Total	Bucharest	Timișoara
Average age (years)	41	39	41
Min (years)	20	20	22
Max (years)	76	63	76
Area of residence			
Rural	33.05%	11.11%	36.2%
Urban	66.95%	88.88%	63.8%
Menopausal status			
Menopause	17.67%	4.44 %	19.55%
Reproductive age	82.33%	95.56%	80.45%

**Table 2:** Indications for hysteroscopy.

	Total ^1^	Bucharest	Timișoara
	(*N* = 368) (100%)	(*N* = 45) (100%)	(*N* = 323) (100%)
IUD retention ^2^	19 (5.16%)	0 (0.00%)	19 (5.88%)
AUB ^3^	131 (35.59%)	36 (80.00%)	95 (29.41%)
Postmenopausal	15 (4.07%)	1 (2.22%)	14 (4.33%)
Endometrial thickening	41 (11.14%)	7 (15.55%)	34 (10.52%)
Hypertrophy	17 (4.61%)	0 (0.00%)	17 (5.26%)
Hyperplasia	24 (6.52%)	7 (15.55%)	17 (5.26%)
Uterine myoma	55 (14.94%)	18 (40.00%)	37 (11.45%)
Intracavitary	19 (5.16%)	1 (2.22%)	18 (5.57%)
Chronic pelvic pain	11 (2.98%)	3 (6.66%)	8 (2.47%)
Adenomyosis	6 (1.63%)	2 (4.44%)	4 (1.23%)
Infertility	68 (18.47%)	7 (15.55%)	61 (18.88%)
primary	36 (9.78%)	2 (4.44%)	34 (10.52%)
secondary	25 (6.79%)	5 (11.11%)	20 (6.19%)
Uterine septum	3 (0.81%)	0 (0.00%)	3 (0.92%)
Polyps	153 (41.57%)	25 (55.55%)	128 (39.62%)
Cervical polyp	12 (3.26%)	4 (8.88%)	8 (2.47%)
Endometrial polyp	140 (38.04%)	21 (46.66%)	119 (36.84%)
Isthmocele	2 (0.54%)	0 (0.00%)	2 (0.61%)

^1^ Data are presented as *N* (%); ^2^ IUD: intrauterine device; ^3^ AUB: abnormal uterine bleeding.

**Table 3:** Hysteroscopic diagnoses by hospital (number of cases).

	Total ^1^	Bucharest	Timișoara
	(*N* = 368) (100%)	(*N* = 45) (100%)	(*N* = 323) (100%)
IUD retention ^2^	16 (4.34%)	0 (0.00%)	16 (4.95%)
Endometrial thickening	63 (17.11%)	16 (35.55%)	47 (14.55%)
Hypertrophy	28 (7.60%)	3 (6.66%)	25 (7.73%)
Hyperplasia	32 (8.69%)	11 (24.44%)	21 (6.50%)
Neoplasia	1 (0.27%)	0 (0.00%)	1 (0.30%)
Uterine myoma	43 (11.68%)	6 (13.33%)	37 (11.45%)
Intracavitary myoma	19 (5.16%)	3 (6.66%)	16 (4.95%)
Adenomyosis	2 (0.54%)	2 (4.44%)	0 (0.00%)
Uterine septum	3 (0.81%)	0 (0.00%)	3 (0.92%)
Synechia	34 (9.23%)	0 (0.00%)	34 (10.52%)
Uterine	26 (7.06%)	0 (0.00%)	26 (8.04%)
Cervical	8 (2.17%)	0 (0.00%)	8 (2.47%)
Polyp	186 (50.54%)	21 (46.66%)	165 (51.08%)
Cervical	16 (4.34%)	4 (8.88%%)	12 (3.71%)
Endometrial	170 (46.19%)	17 (37.77%)	153 (47.36%)
Isthmocele	1 (0.27%)	0 (0.00%)	1 (0.30%)
Normal uterine cavity	34 (9.23%)	1 (2.22%)	33 (10.21%)

^1^ Data are presented as *N* (%); ^2^ IUD: intrauterine device.

**Table 4:** Hysteroscopic procedures by hospital (number of cases).

	Total ^1^	Bucharest	Timișoara
	(*N* = 368) (100%)	(*N* = 45) (100%)	(*N* = 323) (100%)
Diagnostic hysteroscopy	338 (91.84%)	25 (55.55%)	313 (96.90%)
Uterine curettage	97 (26.35%)	23 (51.11%)	74 (22.91%)
Endometrial biopsy	14 (3.80%)	8 (17.77%)	6 (1.85%)
Endometrial resection	13 (3.53%)	0 (0.00%)	13 (4.02%)
Endometrial ablation	3 (0.81%)	0 (0.00%)	3 (0.92%)
Excision of polyps	161 (43.75%)	17 (37.77%)	144 (44.58%)
Fibroid resection	25 (6.79%)	1 (2.22%)	24 (7.43%)
Uterine synechia lysis	7 (1.90%)	1 (2.22%)	6 (1.85%)
IUD extraction ^2^	16 (4.34%)	0 (0.00%)	16 (4.95%)
IUD insertion ^2^	2 (0.54%)	0 (0.00%)	2 (0.61%)
Isthmocele repair	1 (0.27%)	0 (0.00%)	1 (0.30%)

^1^ Data are presented as *N* (%); ^2^ IUD: intrauterine device.

**Table 5:** Histopathological diagnoses by hospital (number of cases).

	Total	Bucharest	Timișoara
	(*N* = 368) (100%)	(*N* = 45) (100%)	(*N* = 323) (100%)
Endometritis	17 (4.61%)	10 (22.22%)	7 (2.16%)
Secretory endometrium	11 (2.98%)	7 (15.55%)	4 (1.23%)
Proliferative endometrium	24 (6.52%)	5 (11.11%)	19 (5.88%)
Simple endometrial hyperplasia	156 (42.39%)	3 (6.66%)	153 (47.36%)
without atypia	49 (13.31%)	1 (2.22%)	48 (14.86%)
with atypia	3 (0.81%)	0 (0.00%)	3 (0.92%)
not specified	104 (28.26%)	2 (4.44%)	102 (31.57%)
Complex endometrial hyperplasia	43 (11.68%)	0 (0.00%)	43 (13.31%)
without atypia	12 (3.26%)	0 (0.00%)	12 (3.71%)
with atypia	10 (2.71%)	0 (0.00%)	10 (3.09%)
not specified	21 (5.70%)	0 (0.00%)	21 (6.50%)
Cervicitis	14 (3.80%)	3 (6.66%)	11 (3.40%)
Polyps	139 (37.77%)	21 (46.66%)	118 (36.53%)
Cervical polyp	7 (1.90%)	2 (4.44%)	5 (1.54%)
Endometrial polyp	132 (35.86%)	19 (42.22%)	113 (34.98%)
Uterine myoma	76 (20.65%)	5 (11.11%)	71 (21.98%)
Adenomyosis	26 (7.06%)	0 (0.00%)	26 (8.04%)
Endometrial neoplasm	7 (1.90%)	1 (2.22%)	6 (1.85%)
Cervical neoplasm	4 (1.08%)	0 (0.00%)	4 (1.23%)
Choriocarcinoma	1 (0.27%)	0 (0.00%)	1 (0.30%)

## Abbreviations

AUB	Abnormal uterine bleeding
IUD	Intrauterine device
HP	Histopathologic
CIN	Cervical intraepithelial neoplasm
FIGO	International Federation of Gynecology and Obstetrics
TV-US	Transvaginal Ultrasound
WHO	World Health Organization
